# The translation research in a dental setting (TRiaDS) programme protocol

**DOI:** 10.1186/1748-5908-5-57

**Published:** 2010-07-20

**Authors:** Jan E Clarkson, Craig R Ramsay, Martin P Eccles, Sandra Eldridge, Jeremy M Grimshaw, Marie Johnston, Susan Michie, Shaun Treweek, Alan Walker, Linda Young, Irene Black, Debbie Bonetti, Heather Cassie, Jill Francis, Gillian MacKenzie, Lorna MacPherson, Lorna McKee, Nigel Pitts, Jim Rennie, Doug Stirling, Colin Tilley, Carole Torgerson, Luke Vale

**Affiliations:** 1 Dental Health Services & Research Unit, University of Dundee, MacKenzie Building, Kirsty Semple Way, Dundee, DD2 4BF, UK; 2 Health Services Research Unit, Health Services Building, University of Aberdeen, Foresterhill, Aberdeen, AB25 2ZD, UK; 3 Institute of Health and Society, 21 Claremont Place, Newcastle University, Newcastle Upon Tyne, NE2 4AA, UK; 4 Institute for Health Sciences Education, Barts and The London School of Medicine and Dentistry, Queen Mary University of London, Abernethy Building, 2 Newark Street, Whitechapel, London, E1 2AT, UK; 5 Ottowa Hospital Research Institute, Administrative Services Building, Room 2-018, 1053 Carling Avenue, Ottawa, K1Y 4EP, Canada; 6 William Guild Building, University of Aberdeen School of Psychology, Aberdeen, AB24 2UB, UK; 7 Centre for Outcomes Research and Effectiveness, Department of Psychology, University College London, 1-19 Torrington Place, London, WC1E 7HB, UK; 8 Division of Clinical & Population Sciences and Education, University of Dundee, Kirsty Semple Way, Dundee, DD2 4BF, UK; 9 NHS Education for Scotland, One Clifton Place, Glasgow, G3 7LD, UK; 10 Scottish Dental Clinical Effectiveness Programme, NHS Education for Scotland, Dundee Dental Education Centre, Frankland Building, Small's Wynd, Dundee, DD1 4HN, UK; 11 University of Glasgow Dental School, 378 Sauchiehall Street, Glasgow, G2 3JX, UK; 12 NHS Education for Scotland, Thistle House, 91 Haymarket Terrace, Edinburgh, EH12 5HE, UK; 13 NHS Education for Scotland, Dundee Dental Education Centre, Frankland Building, Small's Wynd, Dundee, DD1 4HN, UK; 14 School of Education, The University of Birmingham, Birmingham, B15 2TT, UK

## Abstract

**Background:**

It is well documented that the translation of knowledge into clinical practice is a slow and haphazard process. This is no less true for dental healthcare than other types of healthcare. One common policy strategy to help promote knowledge translation is the production of clinical guidance, but it has been demonstrated that the simple publication of guidance is unlikely to optimise practice. Additional knowledge translation interventions have been shown to be effective, but effectiveness varies and much of this variation is unexplained. The need for researchers to move beyond single studies to develop a generalisable, theory based, knowledge translation framework has been identified.

For dentistry in Scotland, the production of clinical guidance is the responsibility of the Scottish Dental Clinical Effectiveness Programme (SDCEP). TRiaDS (Translation Research in a Dental Setting) is a multidisciplinary research collaboration, embedded within the SDCEP guidance development process, which aims to establish a practical evaluative framework for the translation of guidance and to conduct and evaluate a programme of integrated, multi-disciplinary research to enhance the science of knowledge translation.

**Methods:**

Set in General Dental Practice the TRiaDS programmatic evaluation employs a standardised process using optimal methods and theory. For each SDCEP guidance document a diagnostic analysis is undertaken alongside the guidance development process. Information is gathered about current dental care activities. Key recommendations and their required behaviours are identified and prioritised. Stakeholder questionnaires and interviews are used to identify and elicit salient beliefs regarding potential barriers and enablers towards the key recommendations and behaviours. Where possible routinely collected data are used to measure compliance with the guidance and to inform decisions about whether a knowledge translation intervention is required. Interventions are theory based and informed by evidence gathered during the diagnostic phase and by prior published evidence. They are evaluated using a range of experimental and quasi-experimental study designs, and data collection continues beyond the end of the intervention to investigate the sustainability of an intervention effect.

**Discussion:**

The TRiaDS programmatic approach is a significant step forward towards the development of a practical, generalisable framework for knowledge translation research. The multidisciplinary composition of the TRiaDS team enables consideration of the individual, organisational and system determinants of professional behaviour change. In addition the embedding of TRiaDS within a national programme of guidance development offers a unique opportunity to inform and influence the guidance development process, and enables TRiaDS to inform dental services practitioners, policy makers and patients on how best to translate national recommendations into routine clinical activities.

## Background

This protocol describes the TRiaDS (Translation Research in a Dental Setting) programmatic approach to the development of a practical evaluative framework for knowledge translation (KT) research. Improvement in the quality of dental care has been a focus of Scottish Government over successive administrations [1,2]. One such initiative was the establishment of the Scottish Dental Clinical Effectiveness Programme (SDCEP) in 2004, to develop user-friendly guidance to promote best practice and improve the quality of dental care in Scotland [2].

A consistent finding in health services research is that the translation of research findings into practice is unpredictable and can be a slow and haphazard process [3]. Studies in medical care in the USA and the Netherlands suggest that 30 to 40% of patients do not receive care according to current scientific evidence, and 20 to 25% of care provided is not needed or potentially harmful [4-6]. A review of quality of care studies from UK primary care concluded that 'in almost all studies the process of care did not reach the standards set out in national guidelines or those set by the researchers themselves' [3]. Evidence about the translation of research findings in dental healthcare identifies similar problems [7].

It is well documented that the translation of guidelines into clinical practice requires more than the publication of evidence-based clinical guidelines [3-7]. There has been increased interest in the scientific study of methods to promote the systematic uptake of research findings into routine clinical practice over the past fifteen years [8-10]. It has been demonstrated that interventions can be effective, but their effectiveness varies across different clinical problems, contexts, and organisations and this variation is, as yet, largely unexplained [11]. Additionally, there are only limited descriptions of the interventions and contextual data, as well as scant theoretical or conceptual rationale for their choice [12]. There is limited understanding of the impact of, and how best to address, potential barriers and enablers to the translation of research into practice [13,14].

As recommended by the Clinical Effectiveness Research Agenda Group (CERAG) [15], KT research must consider the multiple levels at which healthcare is delivered, their interplay, and the impact of context. There is a need for the development of an understanding of the mechanisms of change from both theoretical and empirical perspectives, as well as methodological issues associated with KT research. The challenge for researchers in the KT research field is to develop and evaluate a theory-based approach that moves beyond single evaluation studies to a generalisable framework that incrementally uses data from a series of evaluations to support, in broadly predictable ways, the choice, development, content, delivery, and evaluation of interventions that aim to change professional behaviour. Such a framework should also facilitate the interpretation of behaviour change research results, both in primary studies and in systematic reviews.

While there is an increasing amount of research looking into medical professional behaviour, there is a dearth of examples of translation research in dental settings. One UK study has investigated the effect of audit and feedback and computer-aided learning in primary dental care [16]. Neither intervention was developed using a theoretical framework and neither influenced evidence-based third molar management. Another UK study, the ERUPT trial [17], examined the effect of a specific fee-for-service and of a general education course (implementing evidence-based practice) on the number of fissure sealants placed. The trial found significantly more fissure sealants were placed by GDPs offered fee-for-service compared to current practice (a general capitation award), but no statistically significant effect of the education intervention. The study contributed to the incentives in healthcare provision debate and led to a policy change with the introduction of a direct fee for this treatment. General dental services are complex small businesses providing a mixture of NHS and private dental care. Although dental practices are subject to regulatory requirements, there is considerable variation in how these are implemented. Therefore, dental practice in Scotland provides the ideal setting for translation research, with generalisable features across other healthcare services, and the opportunity to influence policy is real.

Efforts to improve the quality of care need to occur at, and be coordinated across, multiple levels such as the patient, clinician, team, organisation and policy [18]. Ferlie and Shortell [19] observed:

'Fuelled by public incidents and growing evidence of deficiencies in care, concern over the quality and outcomes of care has increased in both the United Kingdom and the United States. Both countries have launched a number of initiatives to deal with these issues. These initiatives are unlikely to achieve their objectives without explicit consideration of the *multilevel approach to change that includes the individual, group/team, organization, and larger environment/system level*. Attention must be given to issues of leadership, culture, team development, and information technology at all levels. A number of contingent factors influence these efforts in both countries, which must each balance a number of *tradeoffs between centralization and decentralization *in efforts to sustain the impetus for quality improvement over time. The multilevel change framework and associated properties provide a framework for assessing progress along the journey.' (our italics).

### Translation research in a dental setting (TRiaDS)

Established in 2008, TRiaDS is a multidisciplinary research collaboration that has been formed to develop a programme of KT research embedded within the SDCEP guidance development process; it has public, academic, policy, service, and professional members.

Adapting the Canadian Institutes of Health Research (CIHR) definition [20], we define KT as:

'a dynamic and iterative process that includes the synthesis, dissemination, exchange and ethically sound application of knowledge to improve ... health ..., provide (higher quality), more effective health services and products and strengthen the healthcare system.'

KT aims to bridge the gap between best available evidence and its routine implementation in clinical practice by facilitating exchange between researchers and stakeholders (*e.g*., healthcare professionals, patients, educators and policy makers) [21]. To do so requires both the understanding of and effecting of change at both micro- (team, healthcare professional and patient) and macro- (environment, policy, and organisation) levels.

As a research collaboration TRiaDS aims to develop and evaluate the implementation of strategies to improve KT into dental practice [22], and offers the potential to create a research laboratory for the provision and exchange of evidence-based information between the TRiaDS collaboration, dental healthcare professionals, educators, and policy makers on how best to translate service and educational initiatives into practice.

### Aim of TRiaDS

The aim of TRiaDS is to improve the quality of the dental healthcare of patients in Scotland by establishing a practical evaluative framework for the translation of guidance through the conduct of a multi-disciplinary programme of translation research embedded within SDCEP.

### Programme objectives

TRiaDS programme objectives are:

1. To describe current activities, determinants of behaviour, and the natural history of change in clinical and administrative behaviours in specified areas of dentistry in Scotland.

2. To review and, as necessary, change the routine collection of data to support the evaluation of practice in relation to areas of specific relevance to SDCEP.

3. To develop criteria to determine if intervention is required to improve the quality of care.

4. To develop interventions to generate change in targeted professional behaviour(s), as appropriate.

5. To evaluate the effectiveness, cost effectiveness, and sustainability of a range of KT interventions using experimental and quasi-experimental study designs.

6. To investigate and describe the process of professional behaviour change and the process by which change occurs using an appropriate mix of qualitative and quantitative methods.

7. To synthesise knowledge gained from multiple and sequential behaviour change evaluations using a theoretical framework to build on and improve methodology.

8. Through the conduct of the programme, inform dental healthcare professionals, patients, educators, and policy makers on how to effectively and cost-effectively translate national recommendations into routine clinical activities.

## Methods

### Setting

There are 959 general dental practices in Scotland with 2,546 general dental practitioners (GDPs) working within them [23,24]. The majority work in group practices with, on average, three GDPs per practice working with a practice team of dental nurses, dental hygienists, and administrative staff. Sixteen percent of these practices are training practices providing vocational training for approximately 150 vocational dental practitioners per year. In addition, 831 dentists and associated teams of dental healthcare professionals in the salaried/community dental service and 287 in the hospital dental service are also expected to incorporate SDCEP guidance into both clinical care and training [24].

### SDCEP guidance development process

The TRiaDS programmatic evaluation takes place alongside and informs the development of dental clinical guidance by SDCEP. The process of guidance development is summarised in Additional file 1, Table S1.

### Choice of topics for SDCEP guidance

Any individual, group, or organisation may propose a topic for guidance development by SDCEP by completing and submitting a topic proposal form. The SDCEP steering group and programme development team make an initial assessment of proposed topics and present these for a final decision to the National Dental Advisory Committee, which meets two to three times a year. Current topics within the SDCEP programme are: conscious sedation, decontamination, emergency dental care, drug prescribing, oral health assessment, dental caries in children, and a practice support manual that provides guidance to support dental practice management and organisation. Other topics for guidance development are being considered. The topic selection criteria and how these relate to the current SDCEP guidance topics are described in Table 1.

**Table 1:** SDCEP guidance--topic selection criteria

				Current Guidance Topics						
**Selection Criteria**				**Conscious Sedation**	**Decontamination**	**Emergency Dental Care**	**Drug Prescribing**	**Oral Health Assessment**	**Dental Caries in Children**	**Practice Support Manual**

**1. **	**Is the topic related to:**									

	**a)**	a condition or process associated with significant morbidity or mortality?							X	

	**b) **	interventions or practices that could:								

		i)	significantly improve patient or carers' quality of life?			X			X	

		ii)	reduce avoidable morbidity?			X	X		X	

		iii)	reduce inequalities in health?			X			X	

		iv)	prevent oral and dental disease?					X	X	

	**c) **	a priority for the health service or government?		X	X	X	X	X	X	X

	**d)**	interventions or practices that might have a significant impact on the financial or other resources of the NHS or society in general?			X	X	X			X

	**e)**	interventions that the NHS could stop using without impairing cost-effective patient care?								

**2.**	**Will the proposed guidance help reduce or avoid inappropriate:**									

	**a) **	clinical practice?		X	X	X	X	X	X	X

	**b)**	variation in clinical practice?		X	X	X	X	X	X	X

	**c)**	variation in access to interventions or treatment?				X		X	X	

**3.**	**Will the guidance still be relevant at the expected date of publication?**			X	X	X	X	X	X	X

**4.**	**Are there any other reasons why guidance is urgently needed *e.g*., is there significant public concern?**				X		X	X	X	

Note: These selection criteria were adapted from the NICE and SIGN guidance process and are applied to all topics under consideration

### TRiaDS: The evaluative framework

**Figure 1:**
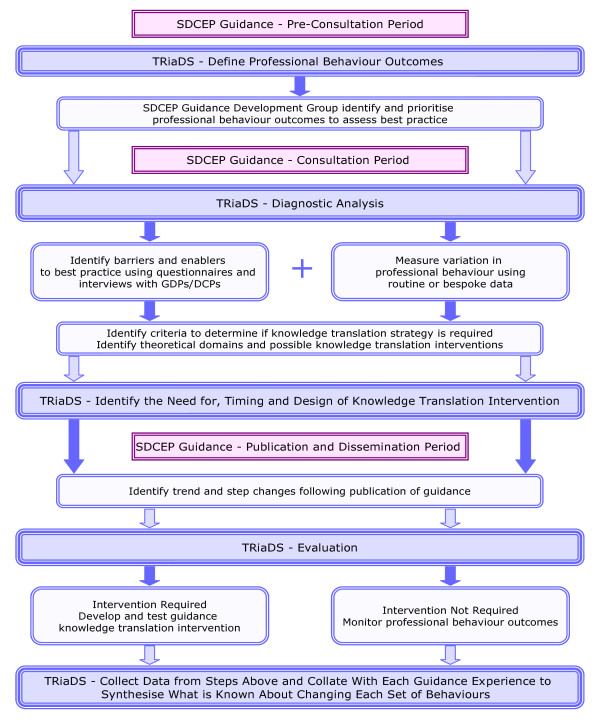
**TRiaDS Framework**.

The programmatic evaluation is a standardised process based on investigations using optimal methods and theory and summarised in Figure 1. For each SDCEP guidance document a diagnostic analysis of relevant current practice commences during the guidance development process. The diagnostic analysis involves gathering information on current dental care activities from a general perspective (*e.g*., the service funding arrangements) and the specific activities/behaviours related to the particular guidance topic (*e.g*., drug prescribing). Where possible, routine data sets such as the Management Information and Dental Accounting System (MIDAS) database (which contains information detailing all NHS Scotland dental treatments provided by GDPs) or the PRISMS drug-prescribing database (which contains information about all encashed NHS Scotland drug prescriptions written by GDPs) are used. If relevant data are not routinely collected, specific data collection tools are developed, piloted, and used. In some cases, some or all of this information will have already been gathered by the SDCEP programme development team as part of the guidance development scoping process, in which case the diagnostic analysis continues this process. However, we anticipate that it will often be necessary to extend beyond the process required for guidance development.

The degree of variation in practice is quantified and information collected to attempt to understand this variation. This process draws information from a range of sources: routinely available data on performance; systematic reviews of the professional behaviour change literature; and focus group and individual interviews with relevant stakeholders (face-to-face or by telephone). These analyses are represented as 'causal maps' with key behaviours, and the links between them identified and set alongside the guidance recommendations.

The diagnostic analysis allows (at least) two questions to be posed: 'What should be done routinely as a consequence of this understanding?' and, 'what should be investigated further as a consequence of this understanding?' An answer to the first question may include monitoring the dissemination of the guidance using routine data. Answers to the second question may include developing complex data collection systems or developing bespoke evaluations.

### Specific processes for evaluating each guidance

A range of evaluative approaches are used. Specific steps that are undertaken for each of the current SDCEP guidance series are detailed below and collated in Table 2. Table 3 summarises TRiaDS' current activities for each of the SDCEP guidance documents.

**Table 2:** Specific processes for evaluating each guidance

SDCEP Activity	TRiaDS Process	TRiaDS Activity		
Pre-consultation period (scoping, evidence, and information retrieval, and appraisal, development of first draft of guidance)	Define professional behaviour outcomes	1.	Collect information from SDCEP guidance development working group and SDCEP Programme Development Team to identify:	
			a)	the key recommendations and required behaviours (what are the behaviours that dental healthcare professionals need to do to follow best practice).
			b)	if these recommendations can be prioritised (what are the most important behaviours in this guidance)?
			c)	the potential barriers and enablers of translation.
	Diagnostic analysis	2.	Use the information to decide on behavioural outcome measures to assess best practice.	
		3.	Identify which of these behavioural outcome measures can be assessed using routinely collected data.	
		4.	If routinely-collected data are not available, determine and develop a bespoke data collection tool.	
		5.	Determine the research feasibility (*e.g*., the costs and benefits relating to associated research requirements, routine or bespoke data collection, intervention, implementation. and evaluation funding).	

Stakeholder consultation period (draft guidance sent to stakeholders (dental healthcare professionals, patients, regulatory and authoritative bodies) for general comments on content, structure, and format of the guidance)	Diagnostic analysis	1.	Conduct telephone interviews/focus groups to identify salient beliefs regarding barriers/facilitators/advantages/disadvantages relating to each behaviour on the outcome list. A random sample of dental health professionals will be invited to take part.	
		2.	Use this information plus stakeholder consultation data to establish:	
			a)	possible predictors of behaviour/behaviour change/theoretical domains relevant to this guidance and identify possible theories which might be used to develop a knowledge translation (KT) intervention if needed.
			b)	the degree of variation in practice.

Pre-publication period (revision, peer review, final amendments)	Decide on the need for and design of KT intervention	1.	Identify criteria to determine if a translation strategy is necessary in total or for each behavioural outcome measure, *e.g*., 50% or 95% adherence to guidance.	
		2.	Test any bespoke tools for gathering non-routinely collected data.	
Dissemination	Decide on the need for and design of KT intervention	1.	Use interrupted time series to identify trend and step changes in routinely available or bespoke data (at least 15 months of data: 12 months pre- and 3 months postguidance consultation/launch/impact on tracer conditions).	
		2.	Survey random sample using self-report questionnaires for data on impact on salient beliefs?	
		3.	Apply identified criteria and determine if an intervention is required.	
Review	Evaluation	1.	Follow specific protocol to develop and test a guidance translation intervention if required	
		2.	Monitor long term guidance outcomes:	
			a)	Develop a universal outcome questionnaire with common and specific questions to each of the published guidance topics. This will be a self-reported tool administered electronically or by post.
			b)	A random sample of dental health professionals will be invited to take part. We will structure the tool for replication within and across guidance topics administered in a block design or universally at an annual or six-month period. An economic analysis will for part of the evaluation of guidance production dissemination and translation.
		3.	Collect data from steps above and collate with each guidance experience (plus the current literature) to quantify (synthesise) what is known about changing each (set of) behaviours (effectiveness of interventions, the process of change, and the predictors of change).	

**Table 3:** TRiaDS process and activity

	Define professional behaviour outcomes	Diagnostic analysis	Decide on the need for and design of knowledge translation intervention	Evaluation
Conscious Sedation				

Decontamination	X	X	X	X

Emergency Dental Care	X	X		

Drug Prescribing	X	X	X	

Oral Health Assessment	X	X		

Dental Caries in Children	X	X		

Practice Support Manual	X			

### Define professional behaviour outcomes

During the pre-stakeholder consultation period, the SDCEP guidance development working group, the SDCEP programme development team, and the TRiaDS team identify the key recommendations and their required behaviours. These are prioritised based on their importance for patient health and/or safety. All, or a subset, of the required behaviours associated with the key recommendations are chosen by the TRiaDS team as the outcomes to be assessed.

### Diagnostic analysis

During the SDCEP stakeholder consultation process, which typically lasts for a period of three months, SDCEP invites stakeholders, such as dental healthcare professionals (*e.g*., GDPs, dental nurses), patients, and regulatory and authoritative bodies (*e.g*., General Dental Council, British Dental Association) to review and comment on the content, structure, and format of the draft guidance document by means of a standardised self-completion questionnaire. In collaboration with SDCEP, TRiaDS incorporates questions to identify current practice and salient beliefs towards the behaviours chosen as the outcomes to be assessed.

In addition, a random sample of dental healthcare professionals is invited to participate in a telephone interview. The interviews follow a standardised structure to identify salient beliefs regarding barriers, facilitators, advantages, and disadvantages that relate to each behavioural outcome [25]. The findings are used to inform intervention design.

Data that could inform judgements about compliance with guidance recommendations are collected from routine sources such as MIDAS and PRISMS. In order for compliance to be assessed before and after the guidance is published, monthly or quarterly data are collected for at least a year prior to consultation until three months post publication. Where routine data do not exist, and the area of practice is judged important, a bespoke data collection exercise is conducted. The bespoke data collection system is generally questionnaire based, but can also include primary data collection within dental practices from practice records, including patients' notes or interviews with the dental team and/or patients.

The quantitative performance data from the diagnostic analysis are analysed using time series designs [26]. This allows an understanding of trends and step changes around events such as a guidance launch.

### Deciding on the need for a KT intervention

Data from the diagnostic analysis allow (at least) four questions to be answered before a decision is made regarding the need for a KT intervention:

1. Do we know that there is suboptimal performance?

2. Do we understand the determinants of behaviour?

3. Can we measure relevant outcomes?

4. Is it feasible to evaluate an intervention (in terms of programme resources and other external factors)?

Criteria for whether an intervention is required include public importance, the size of gap between current professional behaviour and guidance recommendations, the reasons for the gap, and the potential to address the barriers in behaviour. A decision to proceed with an intervention requires, at a minimum, evidence of a gap between current professional behaviour and recommended professional behaviour. Information relating to the expected costs and benefits of the decision to proceed--including obtaining access to routine data, undertaking the diagnostic analysis, and developing the intervention--will also be considered.

Prior to publication of each guidance document, a decision on the timing of an intervention, if required, is made. When current literature and/or synthesised evidence from the TRiaDS evaluative framework suggests publication of the guidance alone is unlikely to change the required behaviour(s), the value of and need for a KT intervention to coincide with publication is considered.

Approximately six months after the publication of the guidance, the need for further intervention or an intervention for all or a proportion of dental health professionals is decided. This decision is based on the interrupted time series analyses of quantitative routinely available or bespoke data (including at least three discrete time points post launch to enable estimation of any trend). The analyses are designed to measure provider-specific variation (to enable non-compliant subgroups to be identified).

### Developing an intervention

The content and method of delivery of an intervention are based on prior published evidence and/or data collected during the diagnostic phase. When there is some urgency to deliver an intervention (*e.g*., if current practice may potentially cause harm), it most likely takes the form of an 'off-the-shelf' intervention based on published research evidence on the effectiveness of the proposed behaviour change intervention, the (cost) effectiveness of the delivery method, and the ease of delivery. An 'off-the-shelf' intervention may also be appropriate where there is less urgency, although such situations offer the opportunity to develop and test tailored theory and evidence-based interventions.

### Evaluating KT interventions

KT interventions are evaluated using experimental or quasi-experimental trial designs. We consider pragmatic cluster randomised designs to be the gold standard. Wherever possible, we would propose using designs that allow evaluation of different KT interventions, such as multi-arm trials or factorial designs. These designs can also incorporate an evaluation of change by the use of baseline measures. In situations where the interventions are intended to be sequentially delivered across all practices, we will consider a stepped-wedge design and randomise the sequence in which practices appear in the delivery process. When randomisation cannot be performed for practical or logistical reasons, interrupted time-series designs will be used. To enhance the time series designs by adjusting for known confounders, TRiaDS will use the methods of instrumental variables recommended by Bloom [27] for evaluating policy interventions, if strong instruments can be identified. If the trials are conducted on a subset of the total population of GDPs in Scotland, the behaviour of non-study participants will also be tracked using routine data when available.

In situations where evaluation using routinely available data is possible, the collection of the data beyond the end of the experimental evaluation continues in order to examine the sustainability of an intervention effect. In addition, a standardised, theoretically-based questionnaire investigates the continued use and impact of SDCEP guidance. This is a self-administered tool, distributed electronically or by post to a random sample of dental health professionals at multiple times annually. An economic analysis of the impact of any change is conducted in parallel.

Whilst TRiaDS' ability to examine unanticipated consequences is limited, it is feasible to routinely monitor the uptake of a number of non-intervention (tracer) conditions [28]. This allows insight into whether activities in one area of guidance appear to have unanticipated consequences in other areas.

## Discussion

The TRiaDS programme is a significant step forward in KT research. To our knowledge, this is one of the first times that a multidisciplinary team has taken forward the challenge of systematically and incrementally developing and evaluating a practical, generalisable framework for KT that incorporates consideration of individual, organisational, and system determinants of professional behaviour change. A similar approach is embedded within the Veterans Affairs Quality Enhancement Research Initiative (QUERI) Programme [29]. Whilst QUERI also works across a healthcare system, it does not have the opportunities offered by a close integration with the guidance development process.

The embedding of TRiaDS within the SDCEP guidance development process offers an outstanding opportunity to shape the guidance development process to promote the translation of the guidance and to prepare the guidance for evaluation. TRiaDS also provides a unique platform to study sustainability. Sustainability is of considerable policy relevance, yet is understudied [15]. Opportunities to study the relative rates of change in intervention and control groups beyond the initial timeframe of an intervention are seldom explored in KT research. Not only does TRiaDS provide policy relevant information, the programme is also a vehicle to address methodological challenges in conducting KT research.

Being embedded within the SDCEP guidance development process with its links to both service and professional bodies enables TRiaDS to inform and to exchange knowledge with dental healthcare professionals, patients, educators, and policy makers on how best to translate national recommendations into routine clinical activities. In addition to the standard academic outputs (papers, conferences), key policy makers are invited at least annually to a TRiaDS meeting for updates, and a briefing report is prepared for stakeholders, including the Chief Dental Officer, Postgraduate Dental Dean and Chair of the National Dental Advisory Committee. The implications for NHS Education for Scotland relates to training in both undergraduate and postgraduate sectors. The findings of TRiaDS also have the potential to inform the development of data collection systems in Scotland through the Scottish Dental Information Group.

Although based in primary dental care in Scotland and centred on clinical guidance for dentistry, the TRiaDS process describes a generalisable, evaluative KT framework that is readily transferable across national and international jurisdictions and professional disciplines.

## Competing interests

Professor Nigel Pitts, in addition to his University roles securing external research grants from Research Councils, the NHS, and commercial funders, serves on a range of advisory panels for dental professional organisations and oral health companies. All other authors declare that they have no competing interests.

## Authors' contributions

All authors contributed to the conceptual design and intellectual content of the framework. JC, CR, ME, SE, JG, MJ, SM, ST, AW, and LY drafted the manuscript. All authors critically reviewed and contributed to draft revisions, and read and approved the final version of this manuscript.

## Supplementary Material

Additional file 1 **Table S1**. SDCEP guidance development process Click here for file
