# Supplementary material for: The translation research in a dental setting (TRiaDS) programme protocol
Source: Implement Sci. 2010 Jul 20;5:57. doi: 10.1186/1748-5908-5-57 (PMC2920875; doi:10.1186/1748-5908-5-57)
Supplement: Additional file 1 — Table S1. SDCEP guidance development process [file 1748-5908-5-57-S1.DOC]

**Table S**1. SDCEP guidance development process

| - Establish a guidance development working group comprising individuals with a particular interest or experience in the topic. - Scope the guidance, which includes the motivation for developing the guidance, the aim of the guidance, what it is to be based on, what it will and will not cover and who the guidance is aimed at (professional and patient groups), and estimation of the potential size of the evidence base. - Evidence and information retrieval, including sourcing existing guidelines, relevant systematic reviews, policy documents, legislation, or other recommendations. These documents are appraised for their quality of development, evidence base, and applicability to the remit of the guidance under development. In the absence of these documents or when supplementary information is required, published literature is searched and unpublished work is sought. - Develop a first draft of the guidance. - Conduct a stakeholder consultation during which the draft guidance is made available for comment. - Revise guidance based on consultation feedback. - As a means of quality assurance, the revised guidance is sent for peer review to clinical and academic experts in the guidance topic. - Final amendments based on feedback from peer review. - Publication in paper form and electronically via the SDCEP website. - Dissemination of published guidance: All GDPs, salaried/community dentists, hospital dentists, and all dental schools in Scotland are sent a copy of the guidance document. In addition, the guidance is sent to relevant UK policy makers, including all four Chief Dental Officers. It is freely available for download from the SDCEP website, and hard copies are sent on request to any dental healthcare professional in the UK. - Review of all aspects of the context of the guidance (regulations, legislation, trends in working practices, and evidence) takes place two years after publication and, if the context has changed significantly, the guidance will be updated accordingly. |
| --- |
